# Selection of Suitable Reference Genes for Normalization of Quantitative Real-Time PCR in Cartilage Tissue Injury and Repair in Rabbits

**DOI:** 10.3390/ijms131114344

**Published:** 2012-11-06

**Authors:** Xiao-Xiang Peng, Rong-Lan Zhao, Wei Song, Hai-Rong Chu, Meng Li, Shu-Ya Song, Guang-Zhou Li, Dong-Chun Liang

**Affiliations:** 1 Shandong Provincial Key Laboratory of Clinical Laboratory Diagnostics, Department of Medical Laboratory, Weifang Medical University, Shandong 261053, China; E-Mails: pxx74@sina.com (X.-X.P.); zhaoronglan@gmail.com (R.-L.Z.); wysw8103@163.com (W.S.); chr7968@163.com (H.-R.C.); 2006limeng@163.com (M.L.); shuyaxin2000@yahoo.com.cn (S.-Y.S.); lgz2006@wfmc.edu.cn (G.-Z.L.); 2 Institute of Endocrinology and Metabolic Disease Hospital, Tianjin Medical University, Tianjin 300070, China; 3 Key Lab of Hormone and Development, Tianjin Ministry, Tianjin 300070, China

**Keywords:** reference gene, quantitative real-time PCR, cartilage tissue, geNorm, NormFinder

## Abstract

When studying the altered expression of genes associated with cartilage regeneration by quantitative real-time RT-PCR (RT-qPCR), reference genes with highly stable expression during different stages of chondrocyte developmental are necessary to normalize gene expression accurately. Until now, no reports evaluating expression changes of commonly used reference genes in rabbit articular cartilage have been published. In this study, defects were made in rabbit articular cartilage, with or without insulin-like growth factor 1 (IGF-1) treatment, to create different chondrocyte living environments. The stability and intensity of the expressions of the candidate reference genes *glyceraldehyde-3-phosphate dehydrogenase* (*GAPDH*), *18S Ribosomal RNA* (*18S rRNA*), *cyclophilin* (*CYP*), *hypoxanthine phosphoribosyl transferase* (*HPRT1*), and *β-2-microglobulin* (*B2M)* were evaluated. The data were analyzed by geNorm and NormFinder. *B2M* and *18S rRNA* were identified to be suitable reference genes for rabbit cartilage tissues.

## 1. Introduction

Many studies have focused on the process of repairing articular cartilage injuries. The various genes involved in this process are differentially expressed at different stages of repair. Quantifying their expression patterns may help us better understand the mechanisms of cartilage regeneration. Quantitative real-time RT-PCR (RT-qPCR) provides the most accurate and specific measure of gene expression [1,2]. However, a number of variables can lead to inconsistent measurements, such as the number and type of cells in the evaluated tissue, the efficiency of mRNA extraction, the mRNA handling techniques [3], the mRNA integrity, the methods of reverse transcription and the analytical detection method [4]. To ensure accurate and specific results, all of these variables must be controlled for thorough normalization, whereby the expression of a target gene is compared to that of a standard reference gene [5,6]. An ideal reference gene should be expressed consistently across all samples being compared, regardless of whether the tissues are collected from physiological or pathological tissues. Housekeeping genes used to be considered to fulfill this criterion and they are the most commonly used reference genes in most experiments.

Normalization by reference genes allows the minimization of variables and increases confidence in relative differences in gene expression. However, more and more evidence suggests that expression of some commonly accepted reference genes, such as *glyceraldehyde-3-phosphate dehydrogenase* (*GAPDH*) and *beta actin* (*ACTB*), are affected by experimental and clinical conditions; therefore, they may not always be suitable controls for normalization [7–10]. Leading qPCR investigators have suggested that reference genes must be validated for each experimental situation to acquire meaningful data [11,12]. Therefore, expression of the prospective reference genes needs to be explored in each specific tissue and type of experiment. In this study, we examined the putative suitability of *GAPDH*, *18S rRNA*, *CYP*, *HPRT1* and *B2M* as reference genes in injury repair of articular cartilage tissue. The five candidate genes tested in this study were chosen based on their previous use as reference genes [13–17]. *ACTB* was not included because the stability of its expression has been challenged in many organisms and tissues [16,18–20]. The RT-qPCR expression data were analyzed using geNorm [21] and NormFinder [22] to identify the most suitable reference genes, namely, those that are minimally affected by chondrocyte development status.

## 2. Results and Discussion

### 2.1. Results

#### 2.1.1. Hematoxylin and Eosin (H and E) Staining

**Figure 1:**
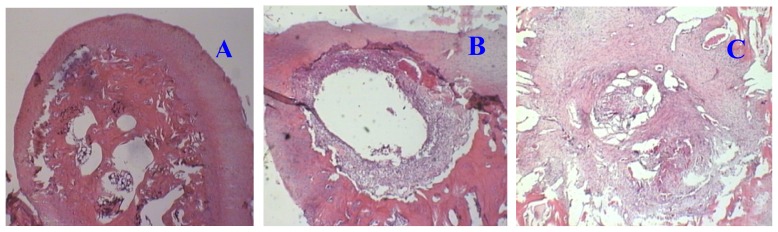
H and E staining of the cartilage sections (40×). The normal control group (**A**) shows normal cartilage structure and composition; Evident cartilage defect could still be observed after 30 days in injury simple group (**B**); rhIGF-1 treatment can markedly enhance the repair of the defect by filling with proliferate chondrocyte and connective tissues (**C**).

H and E staining is shown in Figure 1. Although there is noticeable chondrocyte proliferation, the injured cartilage had a limited capacity for self-repairing (Figure 1B). The defect was minimally repaired or filled after 30 days of recovery; treatment with IGF-1 greatly enhanced the repair of injured cartilage. As shown in Figure 1C, in the IGF-1 treated samples, the defect was filled with proliferative chondrocytes and connective fibers.

#### 2.1.2. Correlation of Reference Genes

**Figure 2:**
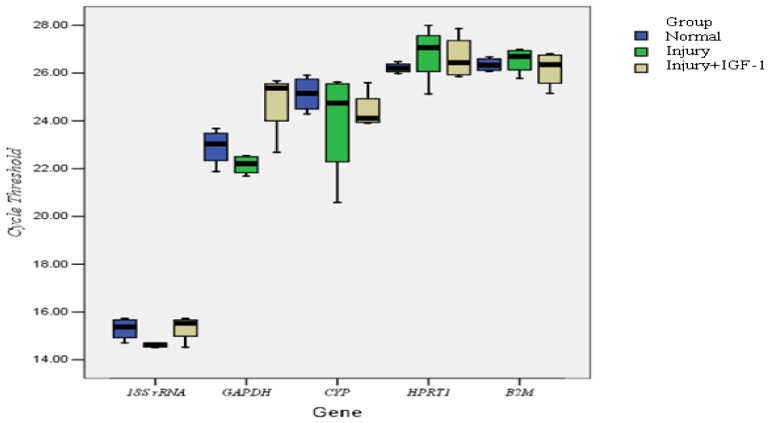
qPCR cycle threshold values for five candidate reference genes among three groups. Candidate reference genes include *18S rRNA*, *GAPDH*, *CYP*, *HPRT1*, and *B2M*. Three groups were evaluated: normal (group A), injury (group B), injury + IGF-1 (group C). Boxes represent lower and upper quartiles of cycle thresholds range with medians. The distributions of *C*t values was wide, from a high of 28.86 (*HPRT1*) to a low of 14.52 (*18S rRNA*).

**Figure 3:**
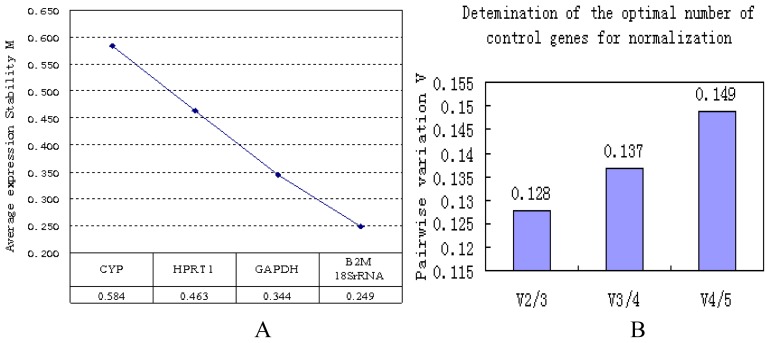
The expression stability of reference genes were evaluated by geNorm. (**A**) Analysis of the average expression stability values (*M*) of the five selected reference genes with geNorm. According to the most stable gene pair to the lowest *M* value, the least stable genes were gradually removed. *B2M* and *18S rRNA* is the gene pair with the lowest *M* value. The *y*-axis indicates the stability parameter M. The *x*-axis from left to right indicates the expression stability of the five selected reference genes; (**B**) The optimal number of normalization reference genes was determined by pair-wise variation analysis. Each bar represents the change in normalization accuracy that occurs when more reference genes are added in a stepwise manner according to the ranking in Figure 3A. To meet the recommended cut-off value of 0.15, two or three genes would be satisfactory for normalization. Based on the pairwise variation results, addition of a third reference gene does not improve normalization in cartilage tissue.

We studied the stability of five candidate reference genes (Table 1) in an articular cartilage injury repair model. Melt curves showed a single melt peak in all reactions except the NTC, which had no amplification. The amplification efficiencies ranged from 1.02 to 1.33. Expression levels among these five candidates varied widely, from highest *C*t of 28.86, for *HPRT1*, to the lowest *C*t of 14.52, for *18S rRNA* (Figure 2). Using the 2 (−Δ*C*t) methods, the *C*t values were transformed into relative quantification data and analyzed using the geNorm and NormFinder programs. geNorm is a statistical algorithm designed to analyze the expression stability of a list of selected reference genes in all samples by calculating M values. In this approach, the mean pairwise variation of a gene compared to all the other reference genes in a given set of samples is called the *M* value. The lower the *M* value, the more stable of expression. We found that the *M* values for the genes (from the most stable to the least stable) were as follows: *B2M* = *18S rRNA* < *GAPDH* < *HPRT1* < *CYP* (Figure 3A). Previous studies had defined *M* = 1.5 as an acceptable cut-off for selection of RT-qPCR reference genes [19,23]. The M values of all the candidates analyzed here are below this cut-off point. However, the *M* values indicated that *CYP* was the least stable reference gene. After stepwise exclusion, the *B2M* and *18S rRNA* gene pair displayed the lowest *M* value, indicating that these two genes are the least affected by the cartilage regeneration status.

The geNorm program also determines a normalization factor (NF) to define the optimal number of reference genes required for an accurate normalization. The NF is calculated from two or more genes with the variable V as the pairwise variation (V*n*/V*n*+1) between two sequential normalization factors (NF*n* and NF*n*+1). We applied the default threshold (0.15) as a cut-off [7]. If the calculated NF is less than 0.15, additional reference genes are not necessary. In all groups evaluated in this study, the pairwise variations were all less than the cut-off threshold (Figure 3B), indicating that using more than two reference genes will not improve the qPCR accuracy. Therefore, the optimal normalization of data in this experimental setting should use two reference genes, *B2M* and*18S rRNA*.

**Figure 4:**
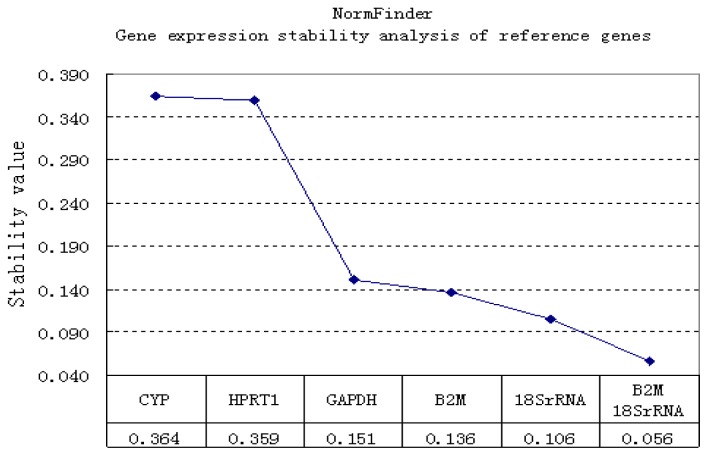
The expression stability of reference genes was evaluated by NormFinder. Five reference genes were used for normalization, and the lowest stability values demonstrate the best gene pair. This figure indicates that *18S rRNA* is the most stable reference gene, while the *B2M* and *18S rRNA* is the most stable gene-pair, namely, the pair with the least variation in gene expression during cartilage tissue injury and repair in rabbits.

The candidate reference genes were also analyzed by another program, NormFinder (Figure 4). In this analysis, higher expression stability is indicated by a lower stability value, which is as an estimate of the combined intra- and intergroup variation of the individual gene [24]. The NormFinder analysis also indicated that *B2M* and *18S rRNA* were the most stably expressed genes. *18S rRNA*, the least affected reference, has a stability value of 0.106. In addition, the geometric average indicated that the combination of *B2M* and *18S rRNA* reduced the stability value to as low as 0.056.

In conclusion, the above results demonstrate that the use of two reference genes (*B2M* and *18S rRNA*) is best for RT-qPCR normalization in rabbit cartilage tissue regeneration studies.

### 2.2. Discussion

RT-qPCR is a highly sensitive method for mRNA quantification in different samples. This technique requires an appropriate normalization of the data to minimize possible differences due to technical variations [21]. Reference genes are commonly used for transcript normalization because of their stable expression [25]. However, a large number of studies show that universal reference genes do not exist, and different experimental methods need to establish their own reference genes to ensure an accurate normalization strategy [23,26–28].

When damaged, the self-repair ability of cartilage tissue is very poor [29]. Cytokines or growth factors are indispensable for augmenting chondrocyte proliferation and cartilage regeneration [30–32]. By local administration, cytokines can increase thickness of the normal cartilage and can accelerate the repair of cartilage defects [33,34]. Cytokines exert their cartilage regeneration-enhancing abilities mostly by stimulating the expression of proliferative genes. In this study, we adopted IGF-1 as the therapeutic factor for repairing cartilage defects, and we applied it to samples at different stages of chondrocyte development to facilitate the determination of the most stably expressed reference genes in regenerating cartilage.

Artificial cartilage defects were induced in groups B and C by surgery, and the repair of defects was detected by histochemistry. H and E staining (Figure 1) confirmed that cartilage with this type of artificial defect had limited self-repair capacity; treatment with IGF-1 markedly stimulated chondrocyte proliferation and the formation of extracellular matrix, indicating that IGF-1 increases the rate of cartilage regeneration. Therefore, the regenerative status of the cartilage was different among the experimental groups.

We analyzed the RT-qPCR data using two different algorithms to determine the optimum number of, and the most stably expressed reference genes.

The geNorm and NormFinder algorithms are widely used and are based on divergent mathematical approaches. geNorm analyzes the expression stability of selected genes in all samples by calculating *M* values (*M*). However, NormFinder evaluates the expression stability of each reference gene independently and takes into account intra- and intergroup variations for normalization [35]. In this study, geNorm (Figure 3) and NormFinder (Figure 4) identified two genes, *B2M* and *18S rRNA*, as the most stable single gene and the most stable reference gene pair to be used for normalizing RT-qPCR data from the cartilage regeneration model. *18S rRNA* has been suggested to be suitable reference gene in different species (*Homo sapiens*, *Rhodnius prolixus* and *Danio rerio*) [20,36,37]. However, some studies have shown that *18S rRNA* is not always a suitable reference gene [9,38]. These different results may be related to the species, tissue, organs or experimental factors. rRNA constitutes the largest fraction of RNA in the cell, and the high abundance of rRNA makes it suitable for high-abundant genes. *B2M* was among the least stable genes in rat MSCs, but was the most stable in rat chondrocytes [39]. Stephens *et al.*[40] reported that *B2M* stably expressed in osteoclasts and macrophages. In addition, we found that *CYP*, *HPRT* and *GAPDH* were unstable in rabbit cartilage tissue. From Figure 4A and NormFinder, it might be concluded that *GAPDH* is as reliable as *B2M*. Here, we show the detailed scores of each program calculation. geNorm analyzes the expression stability by calculating M values, the lower the value of M, the more stable of expression (*GAPDH* 0.344, *B2M* 0.249). With NormFinder, higher expression stability is indicated by a lower stability value (*GAPDH* 0.151, *B2M* 0.136). Together, geNorm and NormFinder showed that *B2M* was superior to *GAPDH* and that *B2M* and *18S rRNA* is the most stable gene pair. Similar results have been shown in human cartilage. Pombo-Suarez M. *et al.*[15] reported that *GAPDH* and *HPRT1* were less stably expressed in human osteoarthritic articular cartilage. Other studies also have shown that *GAPDH* has high expression variance in human mesenchymal stromal cells and hypoxia-cultured human chondrocytes [16,41]. Maccoux *et al.*[42] studied the expression stability of twelve newly identified housekeeping genes in canine osteoarthritic joint tissues. Their results showed that only a small number of the candidates are truly consistently expressed in a certain tissue type. In fact, when different candidate genes are studied, different genes might be identified as the most suitable references. Maccoux *et al.*[42] indicated that *RPS4X* and *RPL13A* are suitable reference genes for dog cartilage. However, their suitability for rabbit chondrocyte needs further investigation. Regardless, what their reports have in common with ours is that theirs also showed that *GAPDH* is less stably expressed than some other reference genes.

Our study shows that *GAPDH* is not suitable as a reference gene for rabbit cartilage even though it had been reported to be suitable for sheep [43]. This agrees with Dundas and Ling [44] and Too and Ling [45] that reference genes suitable for a specific organism or tissue may not be suitable for another organism or tissue. Thus, this paper compared the expression stability of the housekeeping genes in each tissue type under each pathological/physiological will undoubtedly be a good reference. So we recommend that researchers use *B2M* or 1*8S rRNA* to calibrate the expression of their interest genes in studies of regenerating rabbit cartilage.

## 3. Experimental Section

### 3.1. Materials

Trizol total RNA extraction reagent was purchased from Invitrogen (Invitrogen, Carisbad, CA, USA). Quantitative real-time PCR detection kit was purchased from Roche (Roche Ltd, Basel, Switzerland). Recombinant human IGF-1 was ordered from R&D (R&D Systems, Minneapolis, MN, USA). Experimental rabbits were housed in individual cages with standard food and water *ad libitum* under normal conditions. All procedures involving animals were approved by the Animal Care and Use Committee of China.

### 3.2. Injury to Articular Cartilage

Three-month-old New Zealand white rabbits (2.8–3.0 kg) were obtained from VITAL RIVER (Beijing, China). Eighteen rabbits were randomly divided into three major groups (A, B and C). The two hind legs of each rabbit were studied; therefore, each group included 12 tissue samples. Rabbits in group A were not injured and served as controls. Artificial cartilage defects were induced in group B and C rabbits. Surgical procedures were performed under general anesthesia induced by intramuscular injection of ketamine hydrochloride (350 mg/kg body weight) and xylazine (5 mg/kg body weight); general anesthesia was maintained by additional injections at the same dosed every hour as required. After parapatellar arthrotomy, the patella was dislocated laterally, and a 5 mm diameter, 2 mm deep, partially articular cartilage defect was created in the patellar groove of the femur using a dentist drill. Defects were sufficiently lavaged with 0.9% saline, and all skin layers were sutured. Group B rabbits were not given IGF-1 treatment; group C rabbits were given IGF-1 after the operation. Postoperatively, the rabbits were returned to cages and allowed to move freely without splinting. One milliliter of rhIGF-1 (300 μg/mL in PBS) was injected into the articular capsules of the knee joints of group C animals every three days for 30 days, while group B rabbits received injections of PBS. The rabbits were sacrificed under general anesthesia. RNA was isolated from one half of each group and used for RT-qPCR. The remaining samples were subjected to histological evaluation.

### 3.3. Histological Evaluation of Articular Cartilage Injury and Repair

For histological evaluation, the whole defect region, including surrounding cartilage and some hard bone tissue, was dissected and fixed in 10% buffered formalin for 1 week. Specimens were decalcified in 0.5% EDTA solution containing 0.1 M amino-*n*-caproic acid (Sigma Chemical Co., St. Louis, MO, USA) and 0.005 M benzamidine and embedded in paraffin. Four-micrometer sagittal sections of the defect area were collected and stained with hematoxylin and eosin (H and E) according to standard protocol.

### 3.4. RNA Isolation and RT-qPCR Amplification of Reference

Defect surrounded cartilage was carefully trimmed off and immediately put into Trizol reagent. Total RNA was extracted from 1mg cartilage homogenate. The concentration and quality of total RNA were determined using a biological spectrophotometer (Eppendorf, Germany). RNA samples had OD260/280 ratios between 1.7 and 2.0 and OD260/230 ratios below 2.0, reflecting high purity and the absence of protein. The integrity of each total RNA sample was assessed by determining the ratio of 28S/18S ribosomal RNAs after agarose gel electrophoresis. Total RNA (0.5 μg) was used as template in a 20 μL cDNA synthesis reaction (42 °C for 60 min) containing 0.2 μg random hexamer, 200 U of M-MuLV reverse transcriptase (Invitrogen, Carisbad, CA, USA), 20 U Ribonuclease inhibitor and dNTPs at a concentration of 1 mM. Each reverse transcript (0.5 μL) was used as template for PCR amplification. In addition, serial dilutions of the template cDNA were used to confirm reaction efficiency. Five reference genes, *GAPDH*, *18S rRNA*, *CYP*, *HPRT1*, and *B2M*, were evaluated in this study. Primers for each gene are shown in Table 1. Optimum annealing temperatures were determined with gradient PCR prior to qPCR. Nontemplate controls were included for quality control purposes. To compare different RNA transcription levels, the Ct values were compared directly. Ct is the number of cycles required for the detection of the fluorescence signal reaches the set threshold level. Studies have shown that there is an inverse linear relationship between *C*t and the amount of template nucleic acid. As the amount of template increases, the Ct value decreases [13]. Each 20 μL reaction contained 10 μL of 2× SYBR Green Mix, 0.5 μL of a 10 μM solution of each primer, 0.5 μL of cDNA and 8.5 μL of sterile distilled water. The RT-qPCR conditions were as follows: 95 °C for 5 min, followed by 35 cycles of 95 °C for 10 s, 58 °C for 20 s and 72 °C of 30 s. PCR was followed immediately by melting analysis, beginning at 72 °C for 1 min and ramping to 99 °C at a rate of 1 °C per 5 s. Each qPCR assay was run with triplicate technical samples. Relative quantification was performed using Light Cycler Software 1.5.0 (Roche Applied Science). To assess the expression stability of the selected reference genes, the raw Ct values were obtained by averaging the Ct values of the sample collection.

### 3.5. Expression Data Analysis

Data were analyzed after the raw Ct values were transformed into relative quantification data by the ΔCt method. In this study, the relative expression level equal was set to 1 for each gene measured with the minimum *C*t, then, 2^−ΔCt^ was used to represent the relative expression level of the reference genes in the other samples(ΔCt = *C*t value of each sample − minimum *C*t value). These data were further analyzed with the geNorm [21] and NormFinder [22] algorithms. The expression levels of candidate reference genes in the three experimental groups are represented as boxplots. Boxes represent the lower and upper quartiles of the cycle thresholds range with medians. The graph was plotted with SPSS 15.0 software.

## 4. Conclusions

In conclusion, by comparing expressions in rabbit cartilage tissue samples, *B2M* and *18S rRNA* were identified as the most stably expressed reference genes in rabbit cartilage tissues. Furthermore, they are the most suitable gene pair for normalizing qPCR data.

## Figures and Tables

**Table 1:** Primers for five candidate reference genes.

Gene name	Gene symbol	Accession number	Amplicon length (bp)	Primer sequences
*Glyceraldehyde-3-phosphate dehydrogenase*	*GAPDH18S rRNA*	NM_001082253.1	198	Forward: ATCCATTCATTgACCTCCACTAC
				Reverse: gTACTgggCACCAgCATCAC

*18S Ribosomal RNA*	*18S rRNA*	NR_033238.1	167	Forward: ATCAgATACCgTCgTAgTTC

				Reverse: TTCCgTCAATTCCTTTAAg

*Beta-2-microglobulin*	*B2M*	XM_002717921.1	157	Forward: AACgTggAACAgTCAgACC

				Reverse: AgTAATCTCgATCCCATTTC

*Cyclophilin*	*CYP*	ABB45383.1	163	Forward: gggAgAgAgAggATATggATAC

				Reverse: AATgCCATAgTgCTTCAgC

*Hypoxanthine phosphoribosyl transferase*	*HPRT1*	NM_001105671.1	175	Forward: TgATTAgTgATgATgAACCg

				Reverse: CACACAgAgggCTACAATg
